# Propagating Activity in Neocortex, Mediated by Gap Junctions and Modulated by Extracellular Potassium

**DOI:** 10.1523/ENEURO.0387-19.2020

**Published:** 2020-03-11

**Authors:** Christoforos A. Papasavvas, R. Ryley Parrish, Andrew J. Trevelyan

**Affiliations:** Institute of Neuroscience, Newcastle University Medical School, Newcastle upon Tyne NE2 4HH, United Kingdom

**Keywords:** electrotonic, excitability, gap junction, parvalbumin, potassium, seizure

## Abstract

Parvalbumin-expressing interneurons in cortical networks are coupled by gap junctions, forming a syncytium that supports propagating epileptiform discharges, induced by 4-aminopyridine. It remains unclear, however, whether these propagating events occur under more natural states, without pharmacological blockade. In particular, we investigated whether propagation also happens when extracellular K^+^ rises, as is known to occur following intense network activity, such as during seizures. We examined how increasing [K^+^]_o_ affects the likelihood of propagating activity away from a site of focal (200–400 μm) optogenetic activation of parvalbumin-expressing interneurons. Activity was recorded using a linear 16-electrode array placed along layer V of primary visual cortex. At baseline levels of [K^+^]_o_ (3.5 mm), induced activity was recorded only within the illuminated area. However, when [K^+^]_o_ was increased above a threshold level (50th percentile = 8.0 mm; interquartile range = 7.5–9.5 mm), time-locked, fast-spiking unit activity, indicative of parvalbumin-expressing interneuron firing, was also recorded outside the illuminated area, propagating at 59.1 mm/s. The propagating unit activity was unaffected by blockade of GABAergic synaptic transmission, but it was modulated by glutamatergic blockers, and was reduced, and in most cases prevented altogether, by pharmacological blockade of gap junctions, achieved by any of the following three different drugs: quinine, mefloquine, or carbenoxolone. Washout of quinine rapidly re-established the pattern of propagating activity. Computer simulations show qualitative differences between propagating discharges in high [K^+^]_o_ and 4-aminopyridine, arising from differences in the electrotonic effects of these two manipulations. These interneuronal syncytial interactions are likely to affect the complex electrographic dynamics of seizures, once [K^+^]_o_ is raised above this threshold level.

## Significance Statement

We demonstrate the spatially extended propagation of activity through a gap junction-mediated syncytium of parvalbumin-expressing interneurons, in conditions that are known to exist at times within the brain. Previous work has only shown gap junction coordination very locally, through directly connected cells, or induced at a distance by pharmacological means. We show that cell class-specific spread is facilitated by raised extracellular K^+^. This is highly pertinent to what happens at the onset of, and during, seizures, when extracellular K^+^ can rise rapidly to levels well in excess of the measured threshold for propagation. Our data suggest that interneuronal coupling will be enhanced at this time, and this has clear implications for the behavior of these cells as seizures progress.

## Introduction

Cortical interneurons are connected by gap junctions to other interneurons within the same class (Galarreta and Hestrin, 1999, 2001; Gibson et al., 1999; Amitai et al., 2002; Hestrin and Galarreta, 2005; Juszczak and Swiergiel, 2009), providing a highly specific, excitatory link between these cells. Initial studies showed that gap junction coupling normalized the voltage difference between two cells and further synchronized their firing with millisecond precision (Galarreta and Hestrin, 1999; Gibson et al., 1999; Bennett and Zukin, 2004; Connors and Long, 2004). These studies, however, only examined very localized sets of interneurons, and the question of how widely this synchronization spreads remained unexamined.

Propagation through this gap junction coupled network has been observed in one particular pathologic condition, in a widely used model of epileptic activity, induced by bathing brain slices in 4-aminopyridine (4-AP; Szente et al., 2002; Gajda et al., 2003; Gigout et al., 2006a). 4-AP blocks various voltage-dependent K^+^ channels and appears to have a disproportionate effect on the population of fast-spiking interneurons, inducing rhythmic bursting in these cells even in the absence of any glutamatergic drive (Avoli et al., 2002; Bohannon and Hablitz, 2018; Parrish et al., 2018). These epileptiform discharges propagate reliably across brain slices, with a broad and relatively slow wavefront. This though represents a rather specific case, in which the spread of activity through the syncytium is facilitated by two factors related to the K^+^ conductance blockade, with cells being depolarized and also electrotonically more compact, and which may not occur *in vivo*. In contrast, we speculated that gap junction-mediated propagation may also be supported by a different change in the neuronal milieu, that has been demonstrated both *in vivo* and *in vitro*: namely, the raised extracellular potassium ([K^+^]_o_) associated with extreme levels of neuronal activity. We now show that this is indeed the case, although the pattern of spreading activity differs qualitatively from that seen in 4-AP. We used mice that express channelrhodopsin under the parvalbumin (PV) promoter, allowing us to stimulate specifically this population of interneurons in a highly focal manner. We then examined the time-locked activity propagating out from this focus of activation. We show that this propagation is sensitive to, but not dependent on, glutamatergic synaptic activity, but it is abolished by any of three different gap junction blockers. We then explore the differences between the two patterns of spreading activity, induced either by high [K^+^]_o_ or 4-AP, using an extended, multineuron, compartmental model of the interneuronal syncytium.

## Materials and Methods

### Cortical expression of optogenetic proteins

All animal handling and experimentation were performed according to the guidelines laid by the UK Home Office and Animals (Scientific Procedures) Act of 1986 and approved by the Newcastle University Animal Welfare and Ethical Review Body (AWERB #545). Cortical channelrhodopsin-2 (ChR2) expression was achieved by using genetically engineered transgenic mice. Brain slices were prepared from first-generation cross-breeding of homozygous floxed channelrhodopsin mice (B6; 129S-Gt(ROSA)26Sortm32(CAG-COP4*H134R/EYFP)Hze/J; stock #012569, The Jackson Laboratory) with homozygous PV-cre mice [B6; 129P2-Pvalbtm1(cre)Arbr/J; stock #008069, The Jackson Laboratory].

### Preparation of brain slices

Twenty-five young mice (6–12 weeks old) of either sex were killed to prepare the brain slices. They were first anesthetized using ketamine (0.3 ml/30 g) and then perfused with ice-cold sucrose-based artificial CSF (ACSF; NaHCO3 24 mm, KCl 3 mm, NaH2PO4 1.25 mm, sucrose 227.8 mm, glucose 10 mm, MgCl2 4 mm) before the brain was removed to prepare coronal brain slices (400 μm thick). The slices were cut on Leica VT1200 vibratome (Leica Microsystems) in ice-cold oxygenated (95% O_2_/5% CO_2_) ACSF (NaCl 125 mm, NaHCO_3_ 26 mm, glucose 10 mm, KCl 3.5 mm, NaH_2_PO_4_ 1.26 mm, MgCl_2_ 3 mm). After cutting, the slices were transferred to an incubation interface chamber (room temperature) perfused with oxygenated (normal) ACSF (NaCl 125 mm, NaHCO_3_ 26 mm, glucose 10 mm, KCl 3.5 mm, NaH_2_PO_4_ 1.26 mm, CaCl_2_ 2 mm, MgCl_2_ 1 mm) for at least 1 h before transferring them to a recording interface chamber. In the recording interface chamber, the ACSF used was normal ACSF, as described above. The ACSF was perfused at 1.5–2.5 ml/min, and its temperature was kept at 33–36°C. The concentration of extracellular K^+^ was systematically increased during the recording by adding KCl to the perfused ACSF.

### Extracellular recordings

Multichannel extracellular recordings were collected at 25 kHz using a linear 16-channel probe configuration (A16x1-2 mm-100–177, NeuroNexus; electrode separation, 100 μm). This was connected to an ME16-FAI-μPA system and MC-Rack software (Multichannel Systems). The signals were filtered using an analog high-pass filter with a 300 Hz cutoff frequency. Data acquisition was conducted using a 1401–3 Analog-Digital converter (Cambridge Electronic Design) and Spike2 software (Cambridge Electronic Design). The electrode array was placed along layer V in the occipital dorsal area of neocortex, approximately corresponding to primary visual cortex (Dong, 2008). Channelrhodopsin was activated by a 470 nm LED, delivering light through a Nikon Plan Fluor 4× objective (numerical aperture, 0.13), using the patterned illuminator Polygon400 (Mightex). The system was controlled and the patterns were designed through the PolyScan 2 software from the same company. The light intensity was measured at ∼2 mW/mm^2^.

AMPA currents were blocked by bathing in 20 μm NBQX (HelloBio), and NMDA currents were blocked using 50 μm d-APV (Abcam). GABA_A_ receptors were blocked by gabazine (20 μm). We used three different gap junction blockers, namely mefloquine (50 μm) and quinine (100 μm; both from Sigma-Aldrich); and carbenoxolone (100 μm; Tocris Bioscience).

### Multiunit activity analysis

The high-pass-filtered signals from multiple electrodes were analyzed to extract features of the multiunit activity (MUA) at different stages of the experiment. We analyzed channels representing the activity from both inside and outside the stimulation area. We first extracted 120 s epochs from the different experimental stages (e.g., low [K^+^]_o_, high [K^+^]_o_), including six stimulation trials (20 s cycle, 3 s stimulation, 17 s rest). For each experimental stage, the 3 s segments of the stimulation trials were extracted to represent the activity during stimulation. The resting (nonstimulated) firing rates for each pharmacological condition were derived from four 3 s segments, two from just prior to each trial, and two from just after. The firing rate (in spikes per second) was calculated independently for all the different segments (a threshold of four SDs was used for spike detection). Additionally, we measured the rhythmicity of the MUA by determining the timing of each detected spike in regard to the pulse train of stimulation (period of stimulation pulse = 50 ms; 25 ms light ON; 25 ms light OFF). We counted separately all the detected spikes that occurred during the ON phase of the stimulation in each 3 s segment. The rhythmicity of the MUA is defined as the ratio between the number of spikes detected during the ON phase and the total number of spikes in the 3 s segment. Notice that this ratio can range from 0 to 1, with 1 indicating that the spikes have a perfect rhythmicity, which is correlated with the stimulation, whereas 0 indicates that they have an anticorrelated rhythmicity. A value of 0.5, on the other hand, indicates the absence of rhythmicity, with the spikes being uniformly distributed along the 50 ms stimulation period. The MUA rhythmicity was applied for both stimulation and baseline segments. The segments representing activity outside the stimulation area were first shifted circularly for 3 ms before the calculation of their rhythmicity to compensate for the minimal delay that is expected for the activity recorded at least 150 μm away from the stimulation area.

Statistical evaluation of the effect of stimulation and pharmacological manipulations on the MUA was conducted using nonparametric tests. The Wilcoxon rank sum test and its *z*-statistic were used to evaluate the effect of stimulation on the baseline MUA activity. The median difference with 95% confidence interval was used to evaluate the effect of the pharmacological manipulations. We used the DABEST package of estimation statistics for the latter case (Ho et al., 2019).

### Simulations and software accessibility

The model cell consists of the following three compartments: one soma (27 μm length, 29 μm diameter, one segment); and two morphologically and biophysically identical dendrites [left and right; 200 μm length (Fukuda et al., 2006), 0.8 μm diameter (Fukuda and Kosaka, 2003), 10 segments]. The interneuron model used in this study is adapted from the biophysically detailed interneuron model from Konstantoudaki et al., 2014 (ModelDB, accession #168310) and can be found in Extended Data 1. The soma is equipped with the following mechanisms: fast Na^+^ current, A-type K^+^ current, delayed-rectifier K^+^ current, slow K^+^ current, N-type high-threshold activated Ca^2+^ current, hyperpolarization-activated cation current (*I*_h_), fast afterhyperpolarization K^+^ current, and a Ca^2+^ buffering mechanism. The dendrites are equipped with the following mechanisms: fast Na^+^ current, A-type K^+^ current, and delayed-rectifier K^+^ current. The conductance values used are shown in Table 1. Seventy identical cells are scattered in a virtual slice with dimensions 650 × 150 × 150 μm. Thus, the density of the population is ∼5000 PV interneurons/mm^3^, which is close to the density found in layer V of mouse primary visual cortex

**Figure 11.**
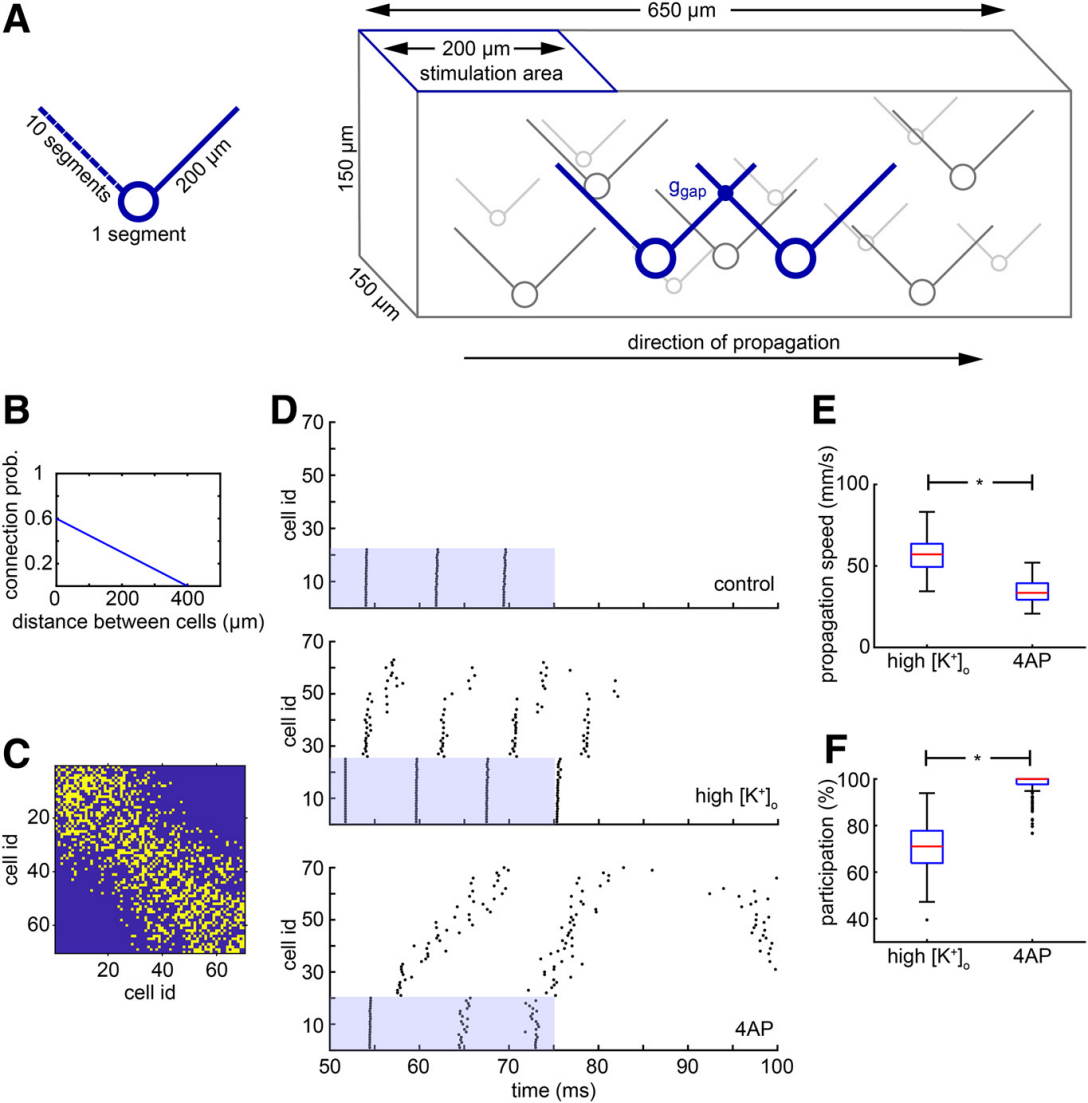
Simulation of the high K^+^ propagation and comparison with the 4-AP propagation. ***A***, Schematic of the model cell and of the model network of electrically connected cells. The cell has a simple morphology with a soma and two dendrites, one on the left and one on the right. The cells are randomly placed in a three-dimensional virtual slice, and they form a network through gap junctions. The left dendrite for each cell is used to connect it with other cells located on its left, whereas the right dendrite is used to connect it with cells on the right. The left side of the network is stimulated and the activity propagates to the right. ***B***, The probability of connection between two cells is linearly decreased as the distance between them increases. ***C***, Example of a connectivity matrix of a randomly generated network where the yellow color indicates a connection. The cell i.d. is derived from the ordering of the cells on the *x*-axis, from left to right. Notice that the leftmost cells do not have any direct connection with the rightmost cells due to the limited length of their dendrite (200 μm). ***D***, Typical results of the simulation under three conditions, as follows: control, high K^+^, and 4-AP. In the control case, there is no propagation; only the cells in the stimulation area fire. In the high K^+^ case, there is a fast propagation to the right side of the network, but not all cells are participating. In the 4-AP case, there is qualitatively different propagation where the speed is lower but almost every cell in the network participates. ***E***, Distribution of propagation speeds for the high K^+^ and 4-AP cases. The propagation under high K^+^ conditions is significantly faster (*p < 0.001, two-sided Wilcoxon rank sum test). ***F***, Distribution of participation percentages for the high K^+^ and 4-AP cases. The participation under 4-AP conditions is significantly higher (*p < 0.001, two-sided Wilcoxon rank sum test). Box plots, indicating the median (red), 25–75th percentiles (blue), and the range extending to 1.5× the interquartile range (whiskers - extreme outliers beyond that range are shown as individual points). The code is available in Extended Data 1.

10.1523/ENEURO.0387-19.2020.ed1 Extended Data 1 Python code for the simulations shown in Figure 11. Download Extended Data 1, ZIP file.

(Pakan et al., 2016). The cells were randomly connected with gap junctions following the connectivity rule (see Fig. 11*B*) A pair of cells would have a maximum of one gap junction (g_gap_ = 0.3 nS; Fukuda et al., 2006; Galarreta and Hestrin, 2001) connecting the right dendrite of the cell on the left with the left dendrite of the cell on the right. The placement of the gap junction along the dendrites was symmetric and was randomly placed with a uniform probability distribution between the closest point possible (considering the distance between the cells) and the full length of the dendrite. For example, two cells with 100 μm distance between them could be connected from the 50 μm dendritic point away from their soma up to the most distal dendritic point, that is, 200 μm away from their soma.

**Table 1:** Biophysical mechanisms used in the model and their conductance values

Mechanism	Soma	Dendrite
Na^+^ conductance (S/cm^2^)	0.045	0.06
Delayed rectifier K^+^	0.018	0.009
N-type Ca^++^	0.0003	N/A
D-type K^+^	0.0000725	N/A
H-current	0.00001	N/A
A-type K^+^	0.048	0.48
fAHP	0.0001	N/A
Ca^++^ diffusion	Yes	No
Cm (μF/cm^2^)	1.2	1.2
Ra (Ω/cm)	150	150
Rm (kΩ cm^2^)	10	10

Extended Data 1. Python code for the simulations shown in Figure 11. fAHP = fast after-hyperpolarization; Cm = membrane capacitance; Ra = axial resistance; Rm = membrane resistance.

The cells that were located in the leftmost 200 μm of the virtual slice were directly stimulated at the soma with a 25 ms pulse of 0.55 nA amplitude starting at 50 ms into the simulation. The speed of propagation is calculated based on the first propagation wave, that is, the first spikes of two specific cells. It is equal to the distance traveled over time between the last cell in the stimulation area and the last cell in the overall propagation. For the results seen in Figure 11, *E* and *F*, only the first 200 long propagations were considered for the analysis. A long propagation is considered a propagation that reaches the 60th cell or beyond that.

All simulations were run using the NEURON simulator (Hines and Carnevale, 2001) through Python (PyNN interface; Davison et al., 2008). A PC running Ubuntu 16.04 LTS was used for the simulations. The code for the simulations described in the paper is freely available online at https://github.com/cpapasavvas/PVsynsytium. The code is available as Extended Data 1.

## Results

### Optogenetic activation of parvalbumin-expressing cells

**Figure 1.**
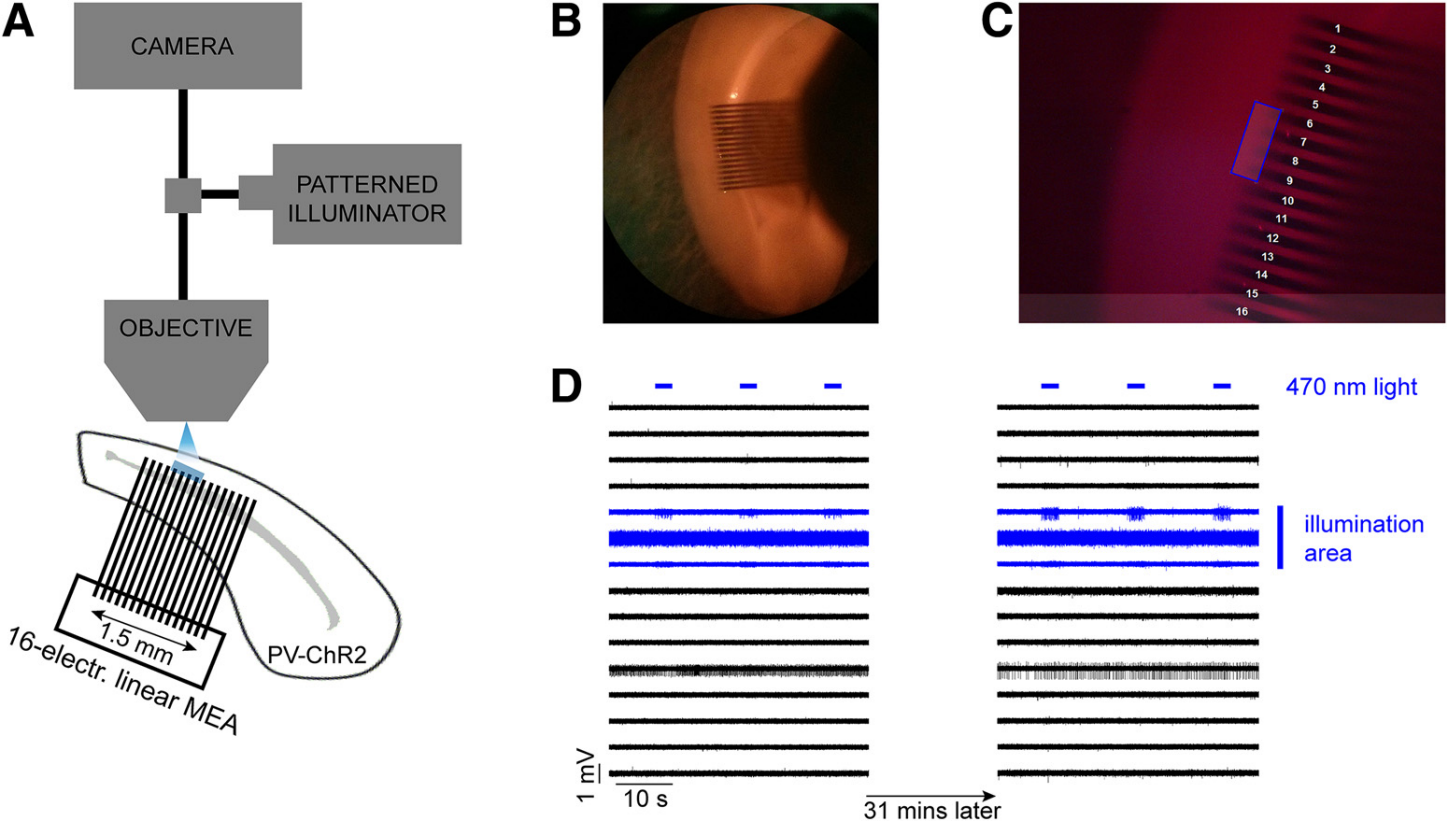
Schematic of the experimental setup and an example of the control experiment. ***A***, Extracellular recordings were taken using a 16-electrode linear MEA placed along layer V in a mouse cortical brain slice in which ChR2 was expressed in PV^+^ cells. The distance between adjacent electrodes was 100 μm. Photostimulation (blue LED) was delivered using a patterned illuminator through the microscope objective. ***B***, The recordings were taken from the dorsal area of the slice, targeting the primary visual area. ***C***, Example of an illumination pattern (four-electrode-wide area) that was drawn in the middle of the array using the patterned illuminator software. ***D***, A typical control experiment during which repeated illumination was delivered for 31 min while the extracellular K^+^ remained at normal levels. The evoked activity was only seen at electrodes within the illumination area, and there was no propagating activity, a pattern that remained stable for the entire duration of the recording (31 min).

We investigated the propagation of activity through the syncytium of PV-expressing interneurons, in occipital cortical brain slices in different levels of extracellular K^+^. Extracellular recordings were made from 53 mouse brain slices prepared from 22 young adult mice that expressed ChR2 under the PV promoter. We recorded extracellular field potentials using a linear multielectrode array (MEA; 1.5 mm wide array of 16 electrodes with 0.1 mm spacing between the shafts) placed along layer V (Fig. 1*B*), where there is a dense network of electrically coupled PV-expressing interneurons (Galarreta and Hestrin, 1999; Gibson et al., 1999; Fukuda and Kosaka, 2003). We used an optogenetic approach to activate only PV-expressing interneurons, in a small and circumscribed area, extending over three to four adjacent electrodes, using a focused patterned illuminator (typically, 300–400 × 100 μm; Fig. 1*C*; and Materials and Methods). The blue light was delivered as a train of pulses lasting 3 s, at 20 Hz frequency with 50% duty cycle, and repeated every 20 s. The spread of activity beyond the light spot was assayed using a linear multielectrode array, which typically extended at least 0.7 mm beyond the light spot, sampling at 100 μm spaces between individual electrodes.

### Propagation of activity with increased extracellular K^+^

**Figure 2.**
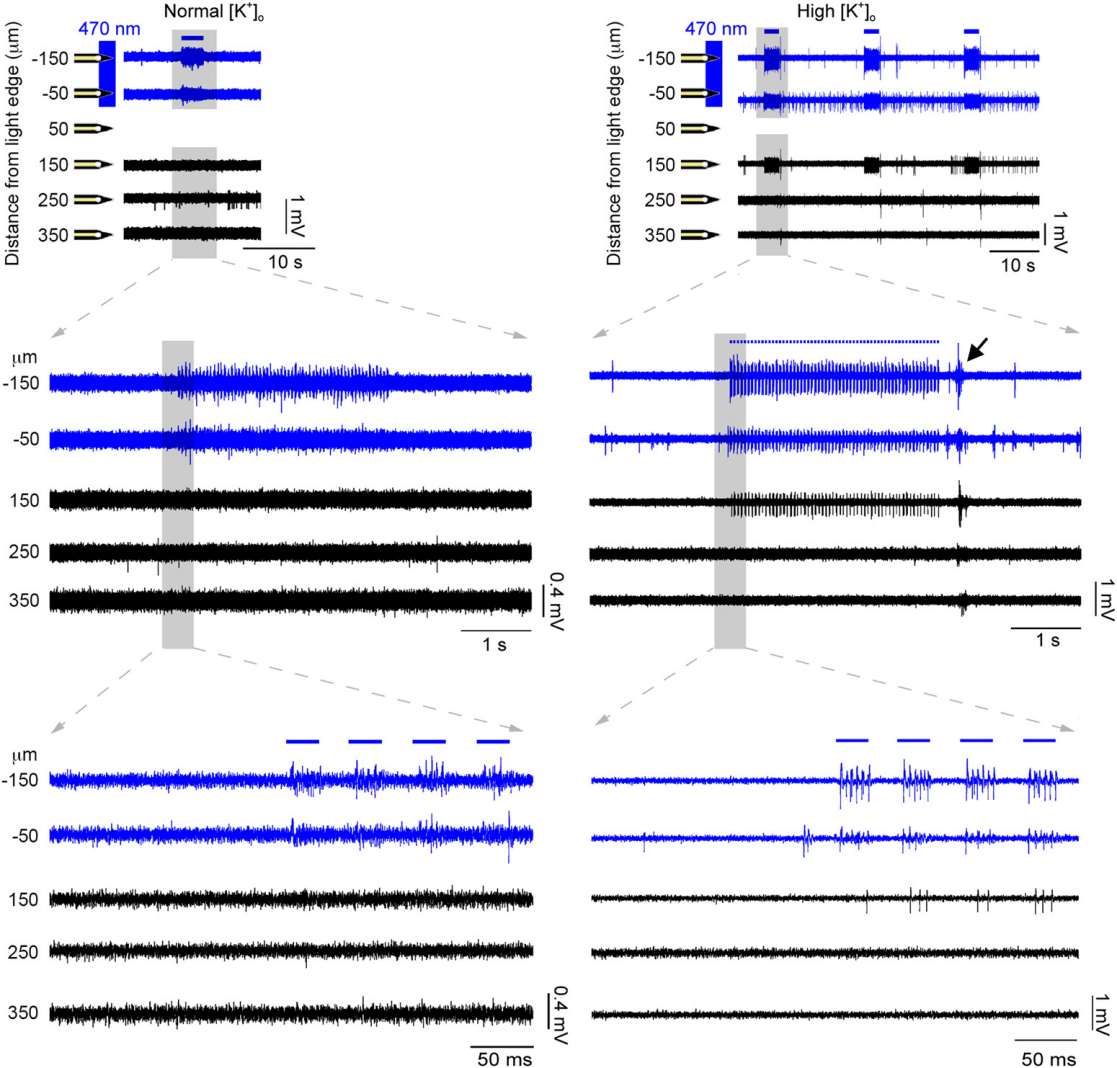
Propagating activity arising with raised extracellular K^+^. The area around a subset of the electrodes was illuminated (marked with blue) with 3-s-long pulse trains (20 Hz, 50% duty cycle), repeated every 20 s. PV^+^ cells around these electrodes responded with four to five spikes per pulse (see zoomed-in traces). After raising the extracellular K^+^ concentration (from 3.5 to 7.5 mm), induced activity was recorded in a distant electrode as well (150 μm away). The activity recorded outside the illumination area was time locked to the activity inside that area, albeit with a short delay. Considering that the photostimulated cells are GABAergic in nature, the activity at the distant electrode is hypothesized to propagate through the electrical synapses between PV^+^ cells. In 6 of the first 12 slices, we saw evidence of rebound bursting at the end of the train of optogenetic stimuli—such an example is seen in the right traces (black arrowhead). Notably, this bursting characteristically involved regular spiking units, and so differed qualitatively from the unit activity seen in the time-locked bursts (Fig. 10).

At baseline levels of extracellular K^+^ ([K^+^]_o_ = 3.5 mm), neuronal spiking was reliably recorded at those electrodes only within the spot of light, or occasionally from electrodes immediately adjacent (50 μm from the edge of illumination), presumably from instances where cells had processes extending into the illuminated area. Critically, though, in this baseline condition, we never recorded triggered activity beyond this restricted site. This pattern of spatially restricted, time-locked activity was very stable, when [K^+^]_o_ was kept constant for an extended period (>30 min, *n* = 3 brain slices, 90 stimulations; Fig. 1*D*). We then increased [K^+^]_o_, with increments of 1-2 mm every 5–10 min, to investigate how this affected activity patterns. Predictably, this produced a marked increase in spontaneous activity (Korn et al., 1987; Jensen and Yaari, 1997), and additionally, in 45.3% of all brain slices (24 of 53 brain slices), we also noted bursts of firing in electrodes away from the illumination site (at least 150 μm away), that were time locked, at short latencies, to the pattern of illumination. Notably, this transition happened rapidly, typically within 2 min, after a threshold level of [K^+^]_o_ was reached, and then remained stable thereafter. Thus, the raised extracellular [K^+^] facilitated the spread of activity away from the focal site of optogenetic activation (Fig. 2).

**Figure 3.**
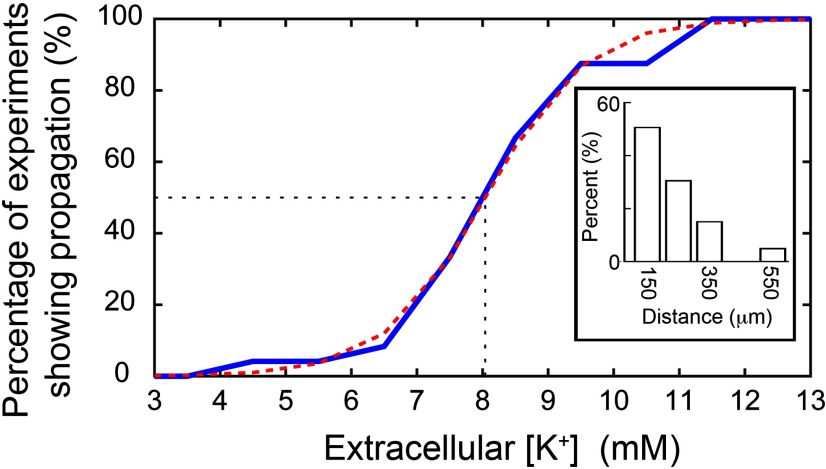
Propagation is facilitated by raised extracellular K^+^ and the spatial spread of the propagations detected. The cumulative proportion of detected propagations at different levels of extracellular K^+^ concentration. The majority of propagations (16 of 24, 66.7%) were observed with an increase of the extracellular K^+^ up to 8.5 mm. The threshold (at the 50% point) was calculated at 8.0 mm after fitting a sigmoidal function (red dashed curve). Inset shows the distance at which time-locked firing was recorded, including instances at up to 550 μm away from the stimulation area.

**Figure 4.**
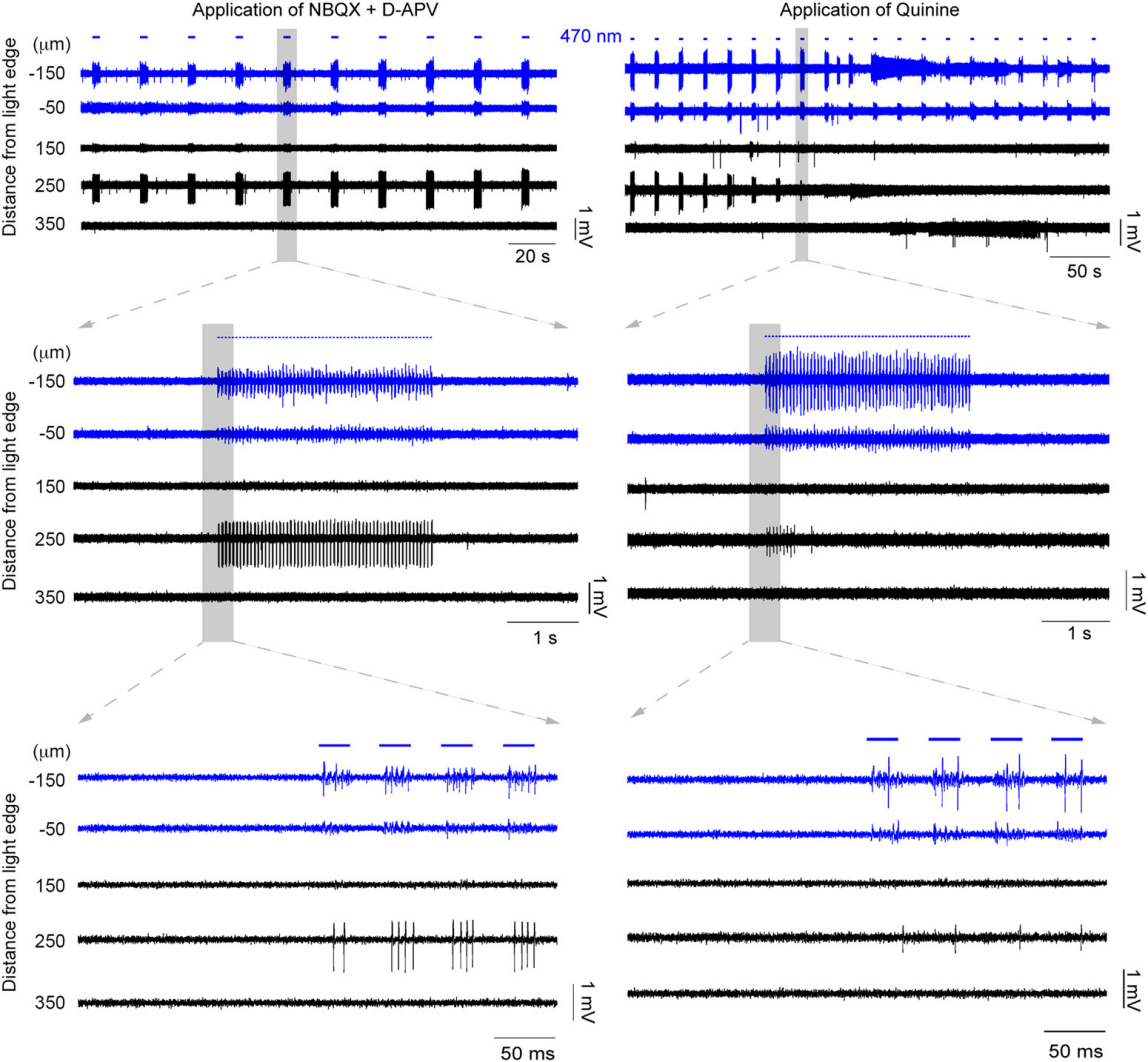
Propagating activity is sensitive to drugs that block glutamatergic receptors and also gap junction blockers. The underlying mechanisms of the propagating activity were investigated by the application of pharmacological agents. This example is the continuation of the example in Fig. 2. First, the glutamate receptors were blocked by applying NBQX and d-APV (left). In this example, the spontaneous activity was decreased but the propagated activity at the distant electrode remained strong. This indicates that glutamate release (e.g., through disinhibition) was not involved in the propagation. Then, the gap junction blocker quinine was applied (right). The activity, recorded 250 μm away, was gradually suppressed, and eventually silenced altogether, as is evident from the decreasing amplitude and spike rate.

The proportion of brain slices that showed propagation away from the point of illumination increased as [K^+^]_o_ was raised (Fig. 3). Time-locked activity was seen most typically in the nearest electrode (150 μm from the edge of illumination), but was found in some cases in electrodes up to 550 μm beyond the edge of the illumination site (Fig. 3, inset). In the more distal cases (≥250 μm from the illumination edge), the majority of cases (9 of 10 brain slices) showed apparent skipping of more proximal electrodes (Fig. 4, example). This we attributed to the sparse distribution of interneurons and the poor sampling of this population by the electrodes. Considering only those slices that showed propagation, we fitted a sigmoidal curve to these data to derive the threshold level of [K^+^]_o_ supporting propagation (50% of trials). The threshold was 8.0 ± 0.15 mm (median ± 95% confidence interval; interquartile range = 7.5–9.5 mm; full range = 4.5–11.5 mm).

### Propagating activity was sensitive to glutamatergic and gap junction blockers, but not GABAergic blockers

**Figure 5.**
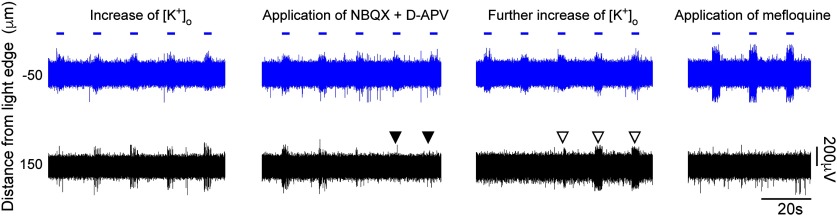
Propagating activity is facilitated by, but is not dependent on, glutamatergic transmission. Repeated propagation assays in the same slice were conducted under different pharmacological conditions. In this example, we first saw propagation at 4.5 mm [K^+^]_o_, but this was suppressed by the addition of glutamatergic blockers (filled arrowheads). Propagating events then resumed once [K^+^]_o_ was increased further to 5.5 mm (open arrowheads). The propagating activity was subsequently blocked entirely by mefloquine.

We hypothesized that the propagating activity spread through gap junction connections between PV-expressing interneurons. Examining only brain slices that showed clear propagation of activity beyond the illumination site (20 slices), and working at increased [K^+^]_o_ (mean = 8.40 mm; range = 4.5–11.5 mm), we first blocked glutamatergic currents using antagonists of AMPA and NMDA receptors (20 μm NBQX and 50 μm d-APV, respectively), to assess what contribution, if any, was made through conventional synaptic excitatory pathways. This also served to reduce the level of spontaneous activity. In a proportion of brain slices (6 of 20 slices), following the introduction of glutamate blockers, the evoked propagating activity gradually diminished in parallel with the reduction in spontaneous activity, indicating that synaptic excitation may contribute to these events (Figs. 4, 5). Notably, in these slices, we were able to resurrect the propagating event by further increasing the extracellular K^+^ (Fig. 5), indicating that propagation is facilitated by glutamatergic activity within the slice, but is not dependent on it. In the remaining brain slices (14 of 20 slices), propagation of activity persisted, following glutamatergic blockade, and indeed was more apparent because it existed on a lower level of background activity.

**Figure 6.**
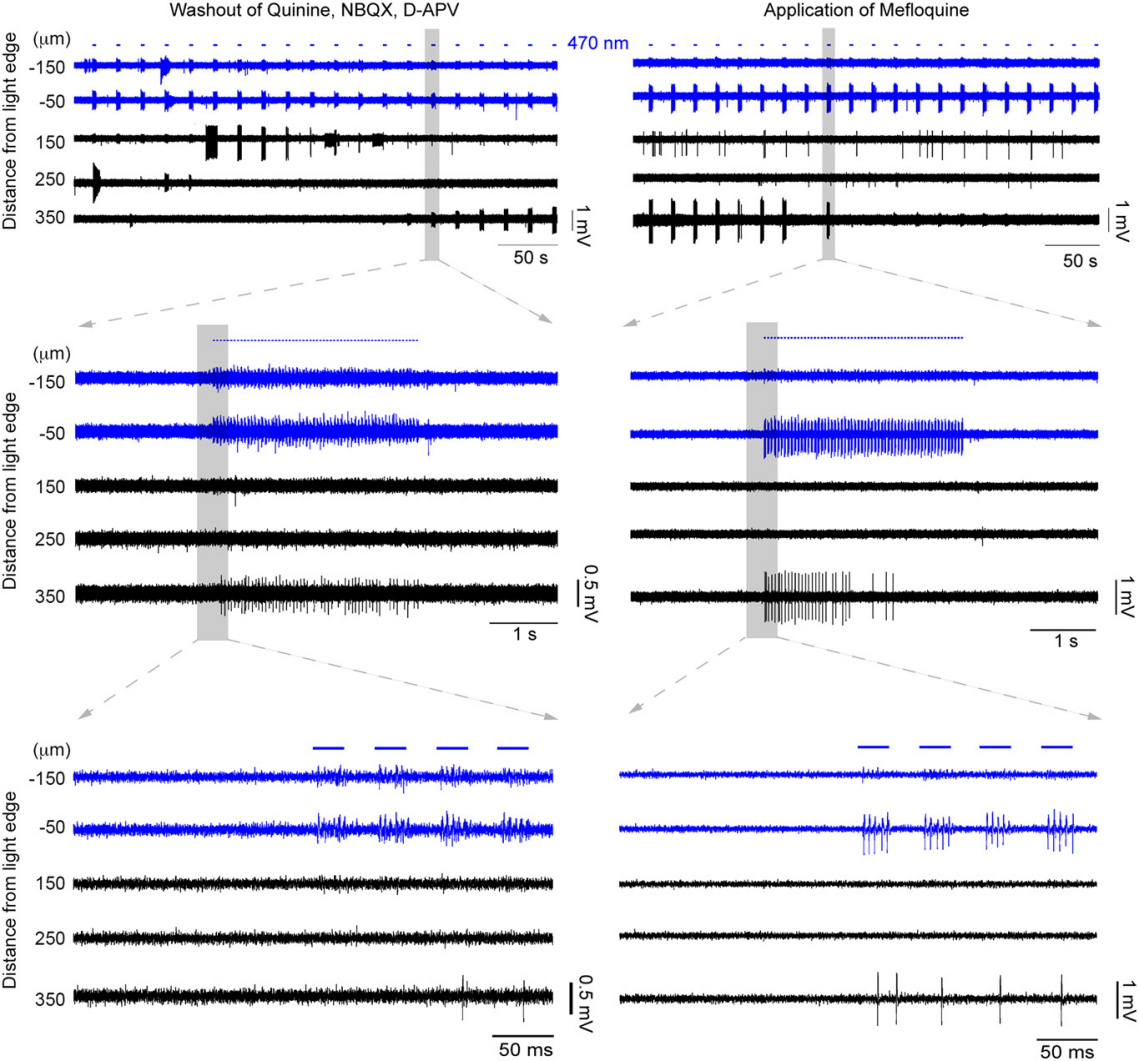
Propagating activity is prevented by multiple different gap junction blockers. The propagating activity silenced in Figure 4 was recovered by washing out the blockers (left). Stable propagating activity with increasing amplitude was recovered at the electrode 350 μm away. This activity remained strong for several minutes until another gap junction blocker was applied, namely mefloquine (right). The propagating activity was once again blocked, validating the involvement of gap junctions by using two different blockers.

We next assessed the effects of gap junction blockers. Unfortunately, this class of drug shows off-target effects (Rozental et al., 2001; Srinivas et al., 2001; Cruikshank et al., 2004), so we examined the following three different gap junction blockers: mefloquine, quinine, and carbenoxolone. In 4 of 14 recordings, propagation persisted 15 min after the application of a gap junction blocker. In the remaining recordings (10 of 14 recordings = 71.4%), propagating activity was abolished (Figs. 4-6, typical examples), and all three drugs showed this effect (quinine, *n* = 3; mefloquine, *n* = 6; carbenoxolone, *n* = 1). The example in Figure 4 shows the effect of quinine, but this pattern appears representative of the other drugs too, illustrating a rapid and marked decrease in both the amplitude and the firing rate of the recorded activity at electrodes away from the illumination site. Importantly, quinine (unlike the other drugs) can be washed out of the bath (Srinivas et al., 2001), and when this was done, propagating activity was rapidly re-established (Fig. 6). When a different gap junction blocker, mefloquine (Figs. 5, 6, right), was then applied, this drug also blocked activity propagation. The recording in Figure 6 further illustrated another principle, that glutamatergic activity did not sustain this pattern of propagation in the absence of gap junction coupling.

**Figure 7.**
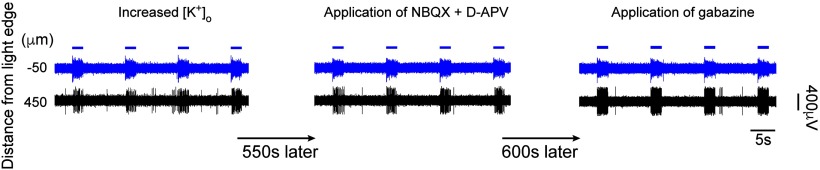
Blockade of GABA_A_ did not block the propagations. In an alternative experimental protocol, GABA_A_ receptors were blocked using gabazine after the blockade of the glutamate receptors. The propagating activity outside the stimulation area remained strong, and the same qualitative result was found in all three slices of this protocol.

In a separate set of experiments, we tested whether the blockade of GABA_A_ receptors affected the propagating activity. In three of seven slices (from three additional mice), we recorded time-locked multiunit activity, under conditions of raised [K^+^]_o_, at sites distant from the illumination locus (150–450 μm from illumination edge). In all three cases, this activity persisted unchanged when we successively applied first glutamatergic blockers and then gabazine to block GABA_A_ receptors. An example trace is shown in Figure 7.

**Figure 8.**
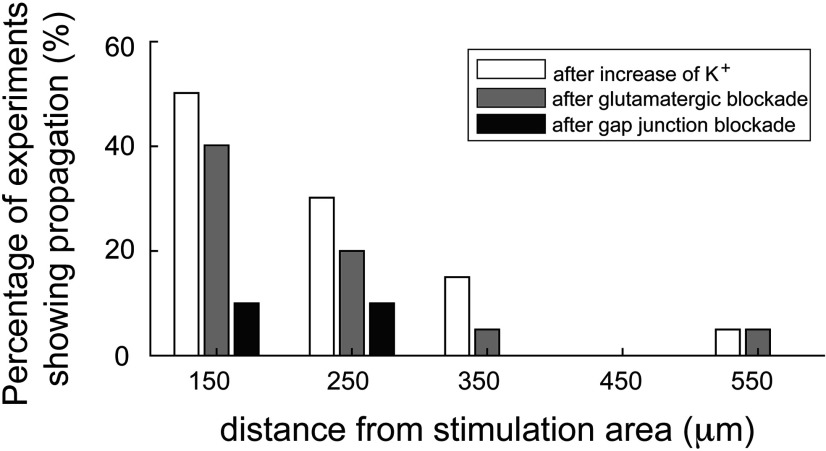
Summary plot of the effects of different pharmacological interventions on the spatial extent of propagating activity.

**Figure 9.**
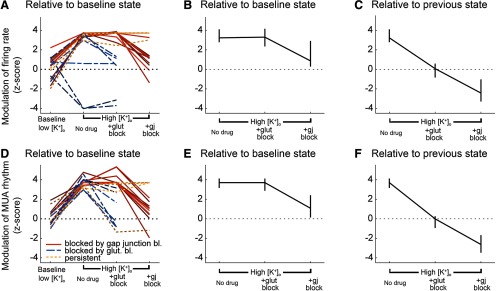
Summary plots of the effects of different pharmacological interventions on multiunit activity (MUA) outside the stimulation area. ***A***, Modulation of the firing rate, by optogenetic stimulation, in different pharmacological conditions. In two recordings, the activity appears to drop significantly during the stimulation. This was actually because these slices had very high baseline activity, but notably, during the stimulation, the activity became tightly time locked in the distal electrodes, indicative of a propagating effect, that was not evident in low K^+^. ***B***, Median *z* score of firing rate modulation, ±95% confidence interval, relative to the baseline condition (3.5 mm [K^+^]_o_). ***C***, Median *z* score of firing rate modulation, ±95% confidence interval, relative to the previous condition. Note that although there were instances of glutamatergic blockade reducing the spread, across all recordings, there was no consistent effect, relative to the prior high [K^+^]_o_ condition. ***D–F***, Equivalent plots showing the modulation of the time-locked rhythmicity in the distant electrodes.

We collated the data from all brain slices that displayed evidence of propagating unit activity induced by raising [K^+^]_o_ (Figs. 8, 9). We analyzed the different pharmacological conditions with respect to the changes in relative activity (Fig. 9*A–C*) and rhythmicity (a measure of the time-locked pattern in Fig. 9*D–F*; see Materials and Methods for further explanation). These analyses show clearly that the propagation is supported in raised [K^+^]_o_ and that it persists following blockade of chemical neurotransmission, but is sensitive to blockade of electrical transmission via gap junctions.

### Propagation involves primarily fast-spiking cells

Gap junctions in PV-expressing interneurons are believed always to connect only to other PV interneurons (Galarreta and Hestrin, 1999; Hestrin and Galarreta, 2005). We reasoned therefore that the analysis of unit spike shapes in the distant electrodes away from the site of stimulation would provide another test of how these events propagate, as follows: events that propagate only through gap junction coupling would show only PV firing at a distance, whereas those that propagate by synaptic means, including glutamatergic or excitatory GABAergic effects, would involve a large degree of pyramidal activation. Spiking in PV interneurons has a highly characteristic signature in the extracellular field potential, with narrow spike widths and prominent overshoot, allowing them to be readily distinguished from so-called “regular-spiking” pyramidal cells.

**Figure 10.**
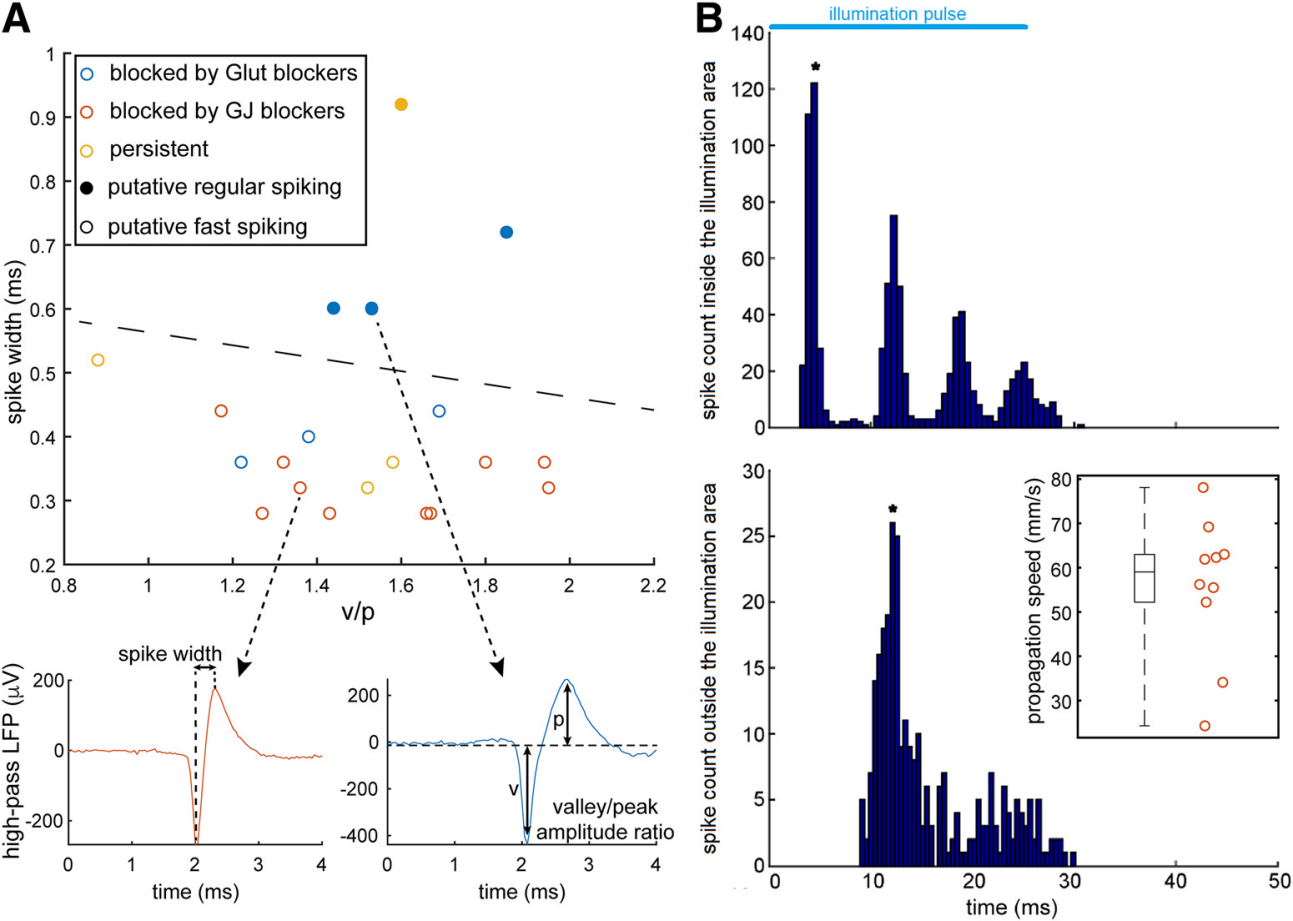
Spike waveform analysis and calculation of propagation speed. ***A***, The spike waveforms of all 20 pharmacologically manipulated propagations were analyzed in terms of their spike width and their amplitude ratio between valley and peak. Both regular- and fast-spiking waveforms were observed (see bottom panels for average spike examples). Notice that none of the hypothesized gap junction-mediated activity was found to exhibit a regular-spiking waveform (i.e., spike width >0.55 ms). ***B***, Example analysis of the speed of propagation calculation, for the gap junction-mediated propagations. The spike time histograms from two different electrodes were plotted: one inside the illumination area (close to the border) and one outside the illumination area where propagating activity was detected. The period covered by the histogram matches the period of the illumination (50 ms). The first peak in each histogram is marked by an asterisk. The propagation speed was directly calculated from the time difference between these peaks and the distance between the respective electrodes. The inset shows the distribution of the calculated propagation speeds (only for propagations blocked by gap junction blockers). It reveals high variability (SD, 17.2 mm/s) and a median speed of 59.1 mm/s.

We separated the 20 brain slices that showed extended propagation into the following three groups: those that were blocked by glutamate blockers (*n* = 6), those that were blocked by gap junction blockers (*n* = 10), and those that persisted after these pharmacological manipulations (*n* = 4). We analyzed the spike waveform of the propagating activity for all three groups. An amplitude-based spike-sorting procedure was applied to filter out any background activity before analyzing the spike waveform of the time-locked propagating activity. Two features were extracted from each spike waveform: its spike width from valley to peak (measured in ms) and the amplitude ratio between valley and peak (Fig. 10*A*, bottom panels; Peyrache et al., 2012). These features are typically used to distinguish activity between fast-spiking and regular-spiking cells (Peyrache et al., 2012). Our spike waveform revealed 4 putative regular-spiking cells and 16 that had a fast-spiking waveform across all groups (Fig. 10*A*).

Notably, in all the brain slices that were blocked by the gap junction blockers (group 2), the units were invariably fast spiking. Pyramidal cells are far more populous than PV interneurons but, on the other hand, usually fire at lower rates. If one assumes that these two effects cancel out, and that therefore one has an equal probability of “finding” a fast-spiking interneuron and a regular-spiking neuron, then the probability of finding just fast-spiking interneurons in every single case (*n* = 10) is ∼0.1%. In spike-sorted data from human neocortex, the ratio of regular-spiking to fast-spiking cells is ∼80:20 (Peyrache et al., 2012), in which case, the probability of our result is orders of magnitude lower. We concluded therefore that finding only fast-spiking neurons, in every brain slice, would have happened only if the propagation were restricted to that cell class, consistent with propagation through the cell class-specific network created by gap junction coupling.

We then analyzed the activity recorded across the multielectrode array with respect to the pulsed timing of the photostimulation (peristimulation spike histograms; Fig. 10*B*), in order to assess the propagation speed of the traveling wave of activity. The speed was calculated from the average latency of the first spikes at distal electrodes. We restricted our analyses only to those experiments where we had pharmacological confirmation of the involvement of gap junctions (i.e., those using quinine, mefloquine, or carbenoxolone). These had a median propagation speed of 59.1 mm/s and a spatial extent of up to 0.55 mm (Fig. 10*B*, inset).

### Simulating propagations through the PV syncytium

The results presented above demonstrate that propagation through the PV-syncytium under conditions of raised extracellular K^+^ is qualitatively different from that induced by 4-AP (Louvel et al., 2001; Gigout et al., 2006a,b). In raised [K^+^]_o_, propagation was significantly faster (59.1 mm/s) than in 4-AP (15 mm/s; Gigout et al., 2006a). There were further differences in the characteristics of the local field potential. In 4-AP, activity had a large low-frequency component, indicative of synchronous activity of many cells, and a broad wavefront (Louvel et al., 2001; Gigout et al., 2006a,b). In high [K^+^]_o_, on the other hand, propagation is manifested as unit activity (single action potentials from isolated cells). As such, the spread of activity in raised [K^+^]_o_ is sparser and is prone to failures of propagation, as evidence showed by the reduced extent of the propagating activity in this model (up to 0.55 mm), compared with 4-AP (>2 mm; Gigout et al., 2006a).

In order to understand these differences better, we developed simulations of biophysically detailed cells that are connected through gap junctions in a three-dimensional virtual slice. In particular, we were keen to assess how the difference in the cellular electrotonic properties in the two cases impacted on the spread. In both cases, neurons are depolarized relative to baseline, but for different reasons: in high [K^+^]_o_, because the K^+^ reversal potential is relatively depolarized; and in 4-AP, because there is a reduced K^+^ conductance. A further difference is that in 4-AP, the blockade of K^+^ channels will additionally reduce the membrane conductivity, meaning that neurons are more electrotonically compact, thus further facilitating spread through the gap junctions.

We modeled each neuron as a simple, three-compartment model, with a soma and two 200-μm-long dendrites (Fig. 11*A*). Seventy of these cells were uniformly scattered in a three-dimensional virtual slice and were randomly connected through gap junctions located on their dendrites (Fig. 11*A*,*B*). Each cell had a “left” and “right” dendrite, and its connectivity with the rest of the network was dictated by its location on the *x*-axis, such that each dendrite only connected to other neurons on that side. This imposed a directionality to the network, so when the left part of the virtual slice is stimulated, the activity propagated from left to right. The cell index (i.d.) for each cell is defined as its rank in the ordered set of cells from left to right [leftmost cell, i.d. = 1; rightmost cell, i.d. = 70]. The connectivity matrix in Figure 11*C* shows an example of a randomly connected network that follows the connectivity rule in Figure 11*B*, and shows that the leftmost cells do not have a direct connection with the rightmost cells.

Cells located in the leftmost 200 μm of the virtual slice were stimulated 50 ms after starting the simulation, and the stimulation pulse, delivered directly to their soma, lasted for 25 ms. The exact number of cells stimulated varied slightly from simulation to simulation since the scattering of the cells in the virtual slice was random. Simulations were run for each of the following three cases: (1) the control case, where the settings are set to default; (2) the high K^+^ case, where the extracellular potassium concentration is considered to be raised to 10.5 mm, instead of the default 3.5 mm, thus changing E_K_ and raising the resting membrane potential, and reducing the effective action potential threshold; and (3) the 4-AP case, where the membrane resistance is five times higher than the default value and the K^+^ channels are almost entirely blocked (conductance is set to 2% of their default value). Typical results of these simulations are shown in Figure 11*D* for each one of the different cases. The blue regions in the scatter plots represent the temporal and spatial extent of the stimulation. For the control case, only the cells in the stimulation area were active, and this activity did not spread beyond the stimulation area at all. In the high [K^+^]_o_ case, activity propagated beyond the area of stimulation, but typically failed before the end of the slice (i.e., 70th cell). Furthermore, we found that even within the propagating territory, the wave of activity could skip some neurons, since there are multiple paths across the network. Thus, not every cell participates in the propagation. Interestingly, the propagation did not advance smoothly. Rather, following stimulation, there was a rapid and almost simultaneous activation of a group of cells in the middle of the slice, followed by a delay before the next set of neurons was recruited.

Gap junction-mediated propagation in the 4-AP simulations, though, was qualitatively different. There was a more gradual and slow propagation that reliably reached the end of the slice without failing. The participation of the cells was complete, with few, if any, being skipped during the propagation (median = 100%; 71.1% for high [K^+^]_o_ simulations; *p* < 0.001, two-sided Wilcoxon rank sum test; Fig. 11*F*). The distribution of the propagation speeds in 200 simulations for each case is shown in Figure 11*E*. The median propagation speed for the high K^+^ case is 57.1 mm/s. This value is close to what was expected from the experimental results presented above. The median propagation speed of the 4-AP case is significantly lower (33.5 mm/s; *p* < 0.001, two-sided Wilcoxon rank sum test) but higher than that in previous studies (15 mm/s; Gigout et al., 2006a).

## Discussion

In these studies, we demonstrate propagating waves of activity within the population of fast-spiking interneurons, extending at distance from the point of onset, at levels of [K^+^]_o_ that are commonly seen during a seizure (Somjen, 2004). Previous work has only shown gap junction coordination very locally, through directly connected cells (Galarreta and Hestrin, 1999; Gibson et al., 1999). Notably, we were able to identify a threshold level of [K^+^]_o_ of ∼8 mm for this propagating activity pattern; below that, at physiological levels of [K^+^]_o_, the activation of PV interneurons remains very focal. Previously, several groups have demonstrated the coordination of interneuronal activation across single gap junctions (Galarreta and Hestrin, 1999; Gibson et al., 1999), but for more extensive propagation within cortical networks, gap junction-facilitated propagation over extended distances has only been demonstrated using pharmacological manipulation, bathing tissue in 4-AP (Szente et al., 2002; Gajda et al., 2003; Gigout et al., 2006a). This pharmacological manipulation, while of interest, does represent a rather extreme disruption of neocortical interneuron behavior (Codadu et al., 2019a). Of particular relevance to the present study is that 4-AP makes neurons more electrotonically compact, by blocking a large component of K^+^ conductance, and will thus naturally facilitate electrotonic propagation. Our new data are the first to show spatially extended propagation in conditions that are known to occur naturally in the living brain.

Since 4-AP has a disproportionately large effect on the population of parvalbumin-expressing interneurons (Codadu et al., 2019a), we also chose to study the effect of raised [K^+^]_o_ in this same population. Electrical coupling between this population of interneurons is well established (Galarreta and Hestrin, 1999; Gibson et al., 1999), but coupling has also been described for other populations of cortical interneurons (Galarreta and Hestrin, 2001; Hestrin and Galarreta, 2005), and our findings are likely to generalize to these interneuronal populations too.

One notable finding is that the pattern of gap junction-coupled propagation in high [K^+^]_o_, where we see tightly time-locked and rapid propagation of single action potentials, appears different from that in 4-AP, which takes the form of a broad wavefront, with a relatively small high-frequency component (Gigout et al., 2006a,b; Parrish et al., 2018). A further distinction between these two experimental paradigms is that 4-AP waves additionally involve other interneuronal populations, and may also trigger waves of raised [K^+^]_o_ themselves, secondary to GABAergic-induced chloride loading (Viitanen et al., 2010), both of which would be expected to broaden the wave. It is noteworthy therefore that 4-AP also blocks K^+^ channels in glia (Hosli et al., 1981), which may boost the transient rise in [K^+^]_o_, an effect that may be further enhanced by gap junction blockers. The resultant slow transient of [K^+^]_o_ following a protracted burst of interneuronal activity (Viitanen et al., 2010) may also contribute to the broad wavefront in 4-AP.

Extended propagation of activity within the PV population only occurred at raised levels of [K^+^]_o_, but appears also to be boosted by background levels of excitatory neurotransmission. Blockade of glutamatergic neurotransmission led to a failure of propagation in a proportion of brain slices, but interestingly, it was possible to resurrect the propagating events by further raising the bathing [K^+^]_o_. Together, these data support the model we present, in which the likelihood of propagation is dictated by the level of depolarization of the cell at rest, which dictates the ease with which the next element in a chain of neurons is recruited, and how electrotonically compact the network is. Both raised [K^+^]_o_ and a tonic level of glutamatergic drive facilitate propagation by the first mechanism, while blocking K^+^ conductance using 4-AP facilitates it by making each element more electrotonically compact. It is possible that excitatory GABAergic activity could also facilitate propagation in this same way, although we did not test this explicitly. It is important though that our results are not consistent with propagation solely through excitatory GABAergic effects, because this would lead to nonspecific activation of different cell classes, whereas our spike-sorting analyses indicate that in the great majority of cases (and all cases in which propagation was suppressed by gap junction blockers), propagating activity is restricted to the fast-spiking class of interneurons. Thus, any contribution from excitatory GABAergic effects is likely only to be an adjunct to the coupling we observed, rather than the primary means of propagation.

The primary effect of enhancing the electrical coupling of interneurons, in this way, will be to extend the inhibitory surround during focal cortical activation (Prince and Wilder, 1967; Schwartz and Bonhoeffer, 2001; Trevelyan et al., 2006; Parrish et al., 2019), while also enhancing the coordination of this inhibitory effect. Interneuronal activity at this time, however, is a double-edged sword, because there is now good evidence that these neurons may become active drivers of epileptic discharges, if pyramidal cells become loaded with chloride (Huberfeld et al., 2007; Dzhala et al., 2010; Avoli and de Curtis, 2011; Ellender et al., 2014; Pallud et al., 2014; Alfonsa et al., 2015, 2016; Raimondo et al., 2015; Chang et al., 2018; Magloire et al., 2019). Raised extracellular K^+^ will also facilitate chloride loading, which is coupled via the cation–chloride cotransporter KCC2. In either case, whether GABAergic output is inhibitory or excitatory, the enhanced coupling of interneurons through their gap junctions is likely to be an important determinant of the complex pattern of propagating local field potentials during a seizure (Viventi et al., 2011; Smith et al., 2016; Codadu et al., 2019b). It is also highly pertinent that gap junction expression is commonly increased in epileptic brains (Manjarrez-Marmolejo and Franco-Pérez, 2016). Whether this is protective, or serves to exacerbate the epileptic condition, remains an open question.

## Synthesis

Reviewing Editor: Alfonso Represa, INSERM Decisions are customarily a result of the Reviewing Editor and the peer reviewers coming together and discussing their recommendations until a consensus is reached. When revisions are invited, a fact-based synthesis statement explaining their decision and outlining what is needed to prepare a revision will be listed below. The following reviewer(s) agreed to reveal their identity: Robert Wykes, Rustem Khazipov.

Dear authors,

Your paper has been evaluated by two reviewers who appreciated the interest of your results that gap-junctions synchronize network of parvalbumin neurons under high potassium conditions. They recommend you however to perform a more completed and detailed description of the spread response, including quantifications of MUA changes evoked by optostimulation over baseline activity at different electrodes, cross-correlation analysis, changes in baseline activity, evoked responses and their spread after pharmacological stimulations and provide statistic evaluations. Thinking on the high synaptic connectivity between PV-cells, referees also propose you to evaluate the effects of pharmacological suppression of GABA(A) receptors on the spread of optostimulation evoked responses.

We also discussed that patch clamp analysis of principal neurons and PV-interneurons, to further evaluate the mechanisms involved on these results, would have greatly improved the quality of the manuscript, but we understand that conducting such experiments would require well over two months of effort and delay the publication of these data. We therefore allow you to consider this suggestion as optional.

Other points raised by referees are:

Figure 1D - 5th black trace from bottom - is this a broken contact?

Rebound burst reliably follows optosimulation response as shown on Figure 2 - was it typical and what could be its mechanism?

Figure 4 - why has activity propagated to 250um, but not 150 or 350um?

Figure 6 - Why is activity in 150um (black) not time-locked with activity −50/−150 (blue)? - In fact looks like it precedes it?

Ln 99-100: sucrose-based solution apparently did not contain sucrose. Please correct.

## Author Response

Dear Alfonso

Thank you for overseeing the editorial process for this paper. We have substantially revised the manuscript, having performed a number more experiments to show that GABAA blockade does not affect the phenomenon, and extending the analyses to address some of the other concerns of the reviewers. To show these, we also include several additional figure panels. We reprint the critiques you sent (we have italicised these for clarity), and we describe the specific changes in response to the particular points.

Your paper has been evaluated by two reviewers who appreciated the interest of your results that gap-junctions synchronize network of parvalbumin neurons under high potassium conditions. They recommend you however to perform a more completed and detailed description of the spread response, including quantifications of MUA changes evoked by optostimulation over baseline activity at different electrodes, cross-correlation analysis, changes in baseline activity, evoked responses and their spread after pharmacological stimulations and provide statistic evaluations.

We have greatly extended the analyses in this regard, and show the new data in an inset in figure 3, and with two new figures (figs 8 and 9). We also extend the descriptions of these analyses in the relevant part of the results section (p10 and p12).

Thinking on the high synaptic connectivity between PV-cells, referees also propose you to evaluate the effects of pharmacological suppression of GABA(A) receptors on the spread of optostimulation evoked responses.

We tried to block the propagating activity using gabazine, and found it had no effect. We show an example set of traces in a new figure (fig 7, and related new passage of text on p12).

We also discussed that patch clamp analysis of principal neurons and PV-interneurons, to further evaluate the mechanisms involved on these results, would have greatly improved the quality of the manuscript, but we understand that conducting such experiments would require well over two months of effort and delay the publication of these data. We therefore allow you to consider this suggestion as optional.

We appreciate this reasonably editorial stance, especially since the two postdocs involved in the study have now both left the laboratory. We agree broadly that such data may indeed have added to the strength of evidence, but we also feel that the phenomenon is robustly demonstrated by the available data, and unfortunately, we do not have the available resources to do these experiments currently.

Figure 1D - 5th black trace from bottom - is this a broken contact?

In fact the opposite - it had what appeared to be a peculiarly active single unit.

Rebound burst reliably follows optosimulation response as shown on Figure 2 - was it typical and what could be its mechanism?

We saw similar bursts in about half the trials. In all of these, notably, the unit activity in the rebound burst was qualitatively different to the optogenetically-modulated activity, in that the rebound burst typically involved predominantly regular-spiking units. We add a sentence describing this difference in the figure legend.

Figure 4 - why has activity propagated to 250um, but not 150 or 350um?

We saw a number of examples such as this, where the activity appears to skip electrodes. We attribute this to a sampling issue, in which the distal time-locked activity indicates that there must have been PV activation in the intermediate territories, but that the electrode simply failed to detect PV units, presumably because their sparse distribution meant that they are not all close to an electrode. Extracellular electrodes will always only detect a tiny proportion of all the active units. We describe this skipping on p10, line 17.

Figure 6 - Why is activity in 150um (black) not time-locked with activity −50/−150 (blue)? - In fact looks like it precedes it?

This particular neuron showed quite a lot of spontaneous bursting activity, and at times, this made it look like it was active ahead of the light. In fact, most times, the time-locked nature of the activity was more evident, and averaged across all trials, this was clearly the dominant pattern. This is shown in the zoomed in version of the relevant part of the figure. Indeed in the right example, the time-locked activity is a different unit from the preceding firing.

Ln 99-100: sucrose-based solution apparently did not contain sucrose. Please correct.

This was a mistake and has been corrected.

Yours sincerely,
